# Temporal metabolic reprogramming in DSS-induced colitis identifies purine metabolism and trigonelline as novel therapeutic targets

**DOI:** 10.3389/fphar.2025.1691978

**Published:** 2025-11-24

**Authors:** Qi Zhang, Jianguo Wang, Long Zhang, Kangjie Song, Xiaoming Sun, Yaming Zhang, Fubao Liu

**Affiliations:** 1 Department of General Surgery, The First Affiliated Hospital of Anhui Medical University, Hefei, China; 2 Department of General Surgery, Anqing Municipal Hospital, Anqing, China; 3 Department of Hepatobiliary Pancreatic Surgery and Minimally Invasive Surgery, Zhejiang Provincial People’s Hospital (Affiliated People’s Hospital), Hangzhou Medical College, Hangzhou, China; 4 Center for Rehabilitation Medicine, Department of Pain Management, Zhejiang Provincial People’s Hospital, Affiliated People’s Hospital, Hangzhou Medical College, Hangzhou, China; 5 Research Institute of Anesthesiology and Perioperative Medicine, Hangzhou Medical College, Hangzhou, China; 6 Department of Gastroenterology, Anqing Municipal Hospital, Anqing, China

**Keywords:** inflammatory, inflammatory bowel disease, metabolomic, trigonelline, purine metabolism

## Abstract

**Background:**

Inflammatory bowel disease (IBD) is a multifactorial disorder characterized by aberrant immune activation and metabolic dysregulation. Despite significant advances in understanding immune mechanisms, the temporal dynamics of metabolic alterations during intestinal inflammation and their therapeutic implications remain poorly defined.

**Methods:**

To investigate metabolic reprogramming during colitis progression, we conducted time-resolved metabolomic profiling of the colon, mesenteric lymph nodes (MLNs), and serum in a dextran sulfate sodium (DSS)-induced murine colitis model at days 1, 3, 5, and 7 post-induction. Targeted and untargeted metabolomic analyses were integrated with pathological and immunological assessments. To assess therapeutic relevance, DSS-treated mice were administered either trigonelline, a metabolite identified in serum, or mycophenolic acid (MPA), a purine metabolism inhibitor, separately. Metabolomic profiling revealed a progressive activation of purine metabolism in colonic tissues and MLNs, correlating with enhanced immune-inflammatory responses.

**Results:**

Trigonelline was identified as a serum biomarker positively associated with disease severity. Therapeutic treatment with either trigonelline or MPA significantly alleviated histopathological damage, reduced inflammatory cell infiltration in both the colon and MLNs, and restored the Th17/Treg cell balance. Mechanistic studies indicated that trigonelline and MPA individually suppress pro-inflammatory signaling pathways while promoting regulatory immune responses.

**Conclusion:**

This study provides a comprehensive temporal map of metabolic reprogramming during colitis progression and identifies purine metabolism and trigonelline as novel therapeutic targets. These findings highlight the translational potential of multi-organ metabolomic approaches in elucidating disease mechanisms and guiding precision treatment strategies for IBD and related inflammatory conditions.

## Highlights

- Time-resolved multi-organ metabolomics maps colitis-associated metabolic dynamics.
- Purine metabolism activation links to intensified immune responses in MLNs and colon.
- Trigonelline emerges as a novel biomarker for colitis progression monitoring.
- Targeting purine metabolism or trigonelline separately alleviates intestinal inflammation.

## 1 Introduction

Inflammatory bowel disease (IBD), comprising ulcerative colitis (UC) and Crohn’s disease, is a chronic relapsing inflammatory disorder of the gastrointestinal tract that presents a growing global health burden (McDowell et al., 2025; Guan, 2019). Despite advances in understanding its immunopathology, the precise mechanisms driving IBD progression remain incompletely defined (Rubin et al., 2012). The dextran sulfate sodium (DSS)-induced colitis model in mice has been widely adopted as a robust and reproducible experimental system that closely mimics the clinical and histopathological features of human UC. This model has proven particularly useful for dissecting host–microbiota–metabolite interactions (Cochran et al., 2020). Emerging evidence highlights the crucial role of metabolites from key pathways, including purine and tryptophan metabolisms, in modulating immune cell function and shaping inflammatory responses in IBD (Yan et al., 2024; Xue et al., 2023; Wang and Zhao, 2025). Nevertheless, the temporal dynamics of these systemic metabolic networks and their interaction with immune dysregulation during the course of colitis remain poorly characterized.

To gain a systems-level understanding of metabolic contributions to intestinal inflammation, we employed a time-resolved, multi-organ metabolomic profiling strategy in a murine model of DSS-induced colitis. Through longitudinal sampling of the colon, mesenteric lymph nodes (MLNs), and serum, we identified dynamic alterations in metabolic pathways associated with the onset and progression of inflammation. Among these, purine and tryptophan metabolisms emerged as key pathways correlating with disease severity. In particular, trigonelline, a tryptophan-derived metabolite with previously reported anti-inflammatory properties, was found to be significantly associated with the inflammatory state (Membrez et al., 2024; Chowdhury et al., 2018).

Tryptophan metabolism plays a central role in immune regulation within the gastrointestinal tract, particularly through its two major branches: the serotonin biosynthetic pathway and the kynurenine pathway (Seo and Kwon, 2023). Inflammatory conditions favor the diversion of tryptophan metabolism toward the kynurenine axis, resulting in the accumulation of immunosuppressive metabolites and the depletion of serotonin, which is involved in epithelial repair and mucosal homeostasis (Tsuji et al., 2023; Sorgdrager et al., 2019; Sukka et al., 2024). Additionally, purine metabolism—through molecules such as adenosine—has been implicated in immune cell modulation and tissue repair during inflammation (Linden et al., 2019; Huang et al., 2021). However, the temporal coordination of these metabolic shifts and their impact on mucosal immunity during colitis remains insufficiently characterized. In this context, we aimed to dissect the metabolic landscape of colitis progression and assess the therapeutic potential of targeting either tryptophan or purine metabolism using trigonelline or mycophenolic acid (MPA), respectively, a selective inhibitor of inosine monophosphate dehydrogenase.

This study advances the understanding of IBD by delineating the metabolic–immune axis in colitis progression and identifying actionable therapeutic targets. We identified several key metabolites, including trigonelline, that are involved in the progression of colitis and may serve as potential biomarkers and therapeutic targets. By targeting key metabolic pathways, such as the tryptophan metabolism and the purine metabolism, we can modulate immune responses and improve disease outcomes in IBD. Our findings highlight the importance of multi-organ metabolomics in unraveling complex disease mechanisms and designing precision therapies for inflammatory disorders.

## 2 Materials and methods

### 2.1 Materials and regents

Trigonelline and mycophenolic acid were purchased from MCE (MedChemExpress). Dextran sulfate sodium (DSS) was purchased from MP Biomedicals (California, United States) (Cat. #9011-18-1, M.W. 36000-50000). The following regents were also used in our study: RPMI 1640 (Gibco, Grand Island, United States) (Cat. #C22400500BT), FBS (Gibco, Grand Island, United States), trypsin (Amresco, United States), EDTA (Invitrogen, United States) (Cat. #2085657), and ELISA kits for TNF-α, IL-6, and IL-17A (R&D Systems, Minneapolis, United States). BV510-conjugated anti-mouse CD4 (BioLegend, United States), FITC-conjugated anti-mouse Foxp3 (BioLegend, United States), PE-conjugated anti-mouse RORγt (BioLegend, United States), eFluor 450 anti-mouse CD326 (EpCAM) Monoclonal Antibody (Clone G8.8, BioLegend, United States), Alexa Fluor® 594 anti-mouse CD326 (EpCAM) antibody (Clone G8.8, BioLegend, United States), Alexa Fluor® 647 mouse anti-Ki-67 (Clone B56, BioLegend, United States), eBioscience™ Fixable Viability Dye eFluor™ 780 (eBioscience™), DNase I (Roche) (Cat. #10104159001), DTT (Merck-Roche), collagenase IV (Sigma-Aldrich) (Cat. #V900893-1g), Precision Count Beads™ (BioLegend)(Cat. #424902), Fixation/Permeabilization Concentrate (Invitrogen) (Cat. #2220750), Percoll^TM^ (GE Healthcare) (Cat. #17-0891-09), ACK lysing buffer (Gibco) (Cat. #A1049201), Triton X-100 (Sangon Biotech) (Cat. #A600198-0500), Tissue-Tek® O.C.T. compound (Sakura Finetek) (Cat. #4583), sucrose (Sigma-Aldrich) (Cat. #v900116), and bovine serum albumin (Sigma-Aldrich) (Cat. #B2064-100G).

### 2.2 Animal models

All animal procedures were conducted in strict accordance with the Guide for the Care and Use of Laboratory Animals published by the U.S. National Institutes of Health (NIH Publication No. 85-23, revised 1996) and were approved by the Ethics Committee of the Zhejiang Academy of Medical Sciences (approval number: ZUCLA-IACUC-20010892). Prior to experimentation, all mice were housed in a specific pathogen–free (SPF) facility under controlled environmental conditions (temperature: 24 °C ± 2 °C; humidity: 60% ± 5%), with a 12-h light/dark cycle. The mice used in this study were C57BL/6 strain, male, aged 8–10 weeks, with an average body weight of 22–26 g at the time of experimentation. There were five mice in each cage, and the mice in the model group and the treatment group were housed in the same cage (e.g., three mice in the intervention group and two mice in the modeling group) to minimize potential cage effects and intestinal flora influences. Animals had *ad libitum* access to standard chow and water throughout the study period. This approach was implemented to ensure consistency and reduce the possibility of cage effects influencing the experimental outcomes.

### 2.3 Colitis model and drug treatment

Experimental colitis was induced by administering 2.5% (w/v) dextran sulfate sodium (DSS; dissolved in distilled drinking water) *ad libitum* for 7 consecutive days, as previously described by Neufert et al. (2007). Body weight was recorded daily for all animals. Mice exhibiting marked weight loss, diarrhea, and visible rectal bleeding by day 3 were considered to have developed colitis and were included for further experimental interventions. Animals were then randomly assigned into three groups (n = 5 per group): a DSS group, a DSS + trigonelline group, and a DSS + mycophenolic acid (MPA) group. Mice in all experimental groups continued to receive 2.5% DSS in drinking water throughout the 7-day experimental period. Trigonelline was administered by oral gavage at a dose of 50 mg/kg body weight daily from day 1 to day 7 following DSS exposure, while MPA was administered via intraperitoneal (i.p.) injection at a dose of 100 mg/kg body weight daily after DSS exposure till tissue collection. The control group received both gavage and i.p. injections of the same-dose vehicles. Trigonelline-treated mice were administered the i.p. vehicle, while MPA-treated mice were gavaged with the vehicle. Trigonelline was administered via oral gavage because it is a naturally occurring dietary alkaloid that is efficiently absorbed through the gastrointestinal tract, reflecting its physiological route of exposure in humans. In contrast, MPA was administered via i.p. injection to ensure precise systemic bioavailability and minimize variability associated with oral absorption, which is known to fluctuate for this compound due to pH sensitivity and first-pass metabolism.

### 2.4 Disease activity index evaluation

Body weight, stool consistency, and fecal blood were monitored daily from the first day of drug administration. The disease activity index (DAI) was assessed as previously described (Xiao et al., 2013), based on a composite scoring system incorporating body weight loss, stool consistency, and the presence of fecal blood. Each parameter was scored according to standardized criteria, and the cumulative DAI score was calculated accordingly. At the end of the experimental period, the colon length of each mouse was measured as an additional indicator of colitis severity.

### 2.5 Measurement of colonic levels of cytokines

Colonic tissues (100 mg) were harvested from each mouse and homogenized using a bead-based homogenizer in 100 μL of RIPA lysis buffer supplemented with a protease inhibitor cocktail. The homogenates were centrifuged at 13,000 rpm for 15 min at 4 °C, and the resulting supernatants were collected as total protein extracts. Protein concentrations were determined using the Pierce BCA Protein Assay Kit (Thermo Fisher Scientific) according to the manufacturer’s instructions.

The expression levels of pro-inflammatory cytokines IL-17A, IL-6, and TNF-α in the tissue homogenates were quantified using enzyme-linked immunosorbent assay (ELISA) kits following the manufacturers’ standard protocols. Cytokine concentrations were normalized to the total tissue weight and expressed as picograms per milligram (pg/mg) of colon tissue.

### 2.6 Sample preparation and metabolome processing

Mice were deeply anesthetized with isoflurane at each time point, and then blood was collected from the heart apex and left on ice for 20 min. Serum was then collected by centrifugation at 12,000 g for 10 min at 4 °C and stored at −80 °C. After blood collection, the mesenteric lymph nodes (MLNs) and colon tissues were harvested immediately, washed with precooled PBS, excess moisture was removed, and the samples were flash-frozen in liquid nitrogen before being stored at −80 °C. Serum (thawed on ice) metabolites were extracted by adding 200 µl methanol at −80 °C to 50 μL of serum sample, followed by vortexing for 5 min, incubation on ice for 10 min, and centrifuging at 4 °C and 12,000 × *g* for 15 min. To extract metabolites from tissue samples, frozen tissue samples were first weighed (∼20 mg each sample) and ground using a homogenizer (Jinxin, Shanghai). Then, they were mixed with 1 mL–20 °C precooled 40:40:20 methanol:acetonitrile:water solution, followed by vortexing for 5 min, incubation at −20 °C for 10 min, and centrifuging at 4 °C and 12,000 g for 15 min. D5-Phenylalanine was added as an internal standard. The supernatant was freeze-dried in a vacuum concentrator (Labconco, CentriVap Benchtop Centrifugal Vacuum Concentrator) at 4 °C, and then dissolved in 50 μL of 50% (v/v, in water) acetonitrile (Sigma-Aldrich) with formic acid, vortexed three times for 30 s, followed by centrifugation at 14,000 × *g* for another 10 min at 4 °C. Then, 5 µL of the supernatant was injected into the liquid chromatography-mass spectroscopy (LC-MS) system(A), with separate injections performed for both positive and negative ionization modes. The widely targeted metabolomics were performed by Wuhan Metware Biotechnology Co., Ltd. (www.metware.cn). In brief, the composition of the resolvent was a mixture of acetonitrile and H_2_O (v:v = 1:1, with 0.03% formic acid). The LC-MS setup consisted of a QTRAP MS (QTRAP 5500, SCIEX) interfaced with a UPLC system (ExionLC AD, SCIEX). Aliquots (2 µL) of each sample were loaded onto a HILIC column (ZIC pHILIC, 5 μm, 2.1 × 100 mm, PN: 1.50462.0001, Millipore). The temperatures of the sampler and column oven were 4 °C and 40 °C, respectively, and the flow rate of the mobile phase was 0.2 mL/min. The mobile phase consisted of 15 mM ammonium acetate containing 3 mL/L ammonium hydroxide (>28%, v/v) in the LC-MS grade water (mobile phase A) and LC-MS grade, 90% (v/v) acetonitrile in LC-MS grade water (mobile phase B). Samples were eluted by a 22-min linear gradient, which began with 95% B and lasted for 1 min, then reached 45% B at 15 min and held for 2 min, and then returned to 95% B at 17.5 min. Metabolites were ionized by electrospray with 3500 V for the positive mode and 2400 V for the negative mode. MS acquisition was performed with a polarity alternating mode. Full MS spectra were acquired from 60 m/z to 900 m/z at 120,000 resolution (at 200 m/z). Data were collected using Analyst software (v.1.7.1, SCIEX), and the relative amounts of metabolites were analyzed using MultiQuant software (v.3.0.3, SCIEX). The area ratio values obtained from the analysis were normalized by statTarget and internal standard corrections. The corrected area ratio values are used for chart presentation. MetaboAnalyst 6.0 (https://www.metaboanalyst.ca/MetaboAnalyst/ModuleView.xhtml) was used to process metabolome data. MetaboAnalyst 6.0 was used to conduct the causal analysis based on Mendelian randomization (MR). Benign neoplasm: The Colon | finn-b-CD2_BENIGN_COLON database was chosen to conduct the mGWAS analysis.

### 2.7 Preparation of cell suspensions, antibody staining, and flow cytometry analysis

Lymphocytes from the colonic lamina propria were isolated using a standardized multi-step enzymatic digestion protocol. In brief, excised colonic tissue segments were first incubated in RPMI 1640 medium (Gibco, Cat. #C22400500BT) supplemented with 0.5 mM EDTA (Sigma-Aldrich, Cat. #2085657) and 0.145 mg/mL dithiothreitol (DTT; Merck-Roche, Cat. #10708984001) at 37 °C for 30 min under constant agitation to remove the epithelial cell layer.

Following epithelial removal, tissues were finely minced and subjected to enzymatic digestion in RPMI 1640 medium containing 10% fetal bovine serum (FBS), 0.5 mg/mL collagenase IV (Sigma-Aldrich, Cat. #V900893-1g), and 100 μg/mL DNase I (Roche, Cat. #10104159001) at 37 °C for an additional 30 min with continuous stirring. The resulting cell suspensions were sequentially filtered through 70-μm and 40-μm nylon cell strainers to eliminate debris. Lymphocytes were subsequently enriched via discontinuous Percoll gradient centrifugation (GE Healthcare, Cat. #17-0891-09).

For flow cytometric analysis, cells were initially stained with fixable viability dyes—either Zombie Red or Fixable Viability Dye eFluor™ 780 (Invitrogen)—for 30 min at 4 °C to exclude non-viable cells. Fc receptors were blocked by incubating cells with anti-mouse CD16/32 antibody (clone 2.4G2, BD Biosciences) for 15 min at 4 °C. Surface marker staining was then performed by incubating cells for 30 min at 4 °C with fluorophore-conjugated antibodies against CD45 (clone 30-F11), CD64, and CD11b (all from BioLegend). After washing, cells were acquired on a flow cytometer for downstream analysis.

### 2.8 Intestinal tissue staining

#### 2.8.1 Immunofluorescence

Immunofluorescence staining and confocal imaging were performed as previously described (Mao et al., 2018), with minor modifications. In brief, colonic and small intestinal tissues were excised and prepared using the Swiss roll technique. Tissues were fixed overnight at 4 °C in BD Cytofix/Cytoperm solution (BD Biosciences, Cat. #554722) diluted 1:2 in phosphate-buffered saline (PBS), followed by dehydration in 30% sucrose for 24 h. Samples were then embedded in O.C.T. compound (Sakura Finetek, Cat# 4583) and cryosectioned into 16-μm slices using a cryostat. Sections were mounted on positively charged slides (Premiere).

Frozen sections were rehydrated in PBS and blocked with a solution containing 1% normal mouse serum, 1% bovine serum albumin (BSA), and 0.3% Triton X-100 in PBS. Staining was performed sequentially by incubating sections with unconjugated primary antibodies overnight at 4 °C, followed by fluorophore-conjugated secondary antibodies for 2–3 h at room temperature, and directly conjugated antibodies for 24 h at room temperature in a dark, humidified chamber. Sections were washed 3–5 times with PBS (5 min per wash) between each staining step. All antibodies were diluted in blocking buffer.

The following antibodies were used: anti-EpCAM, anti-Ki67, anti-CD4, anti-Foxp3, and anti-RORγt. After staining, slides were mounted with Fluoromount-G (SouthernBiotech, Cat# 0100-01), and images were captured using a Leica TCS SP8 confocal microscope. Image processing and quantification were performed using Imaris software (Bitplane).

#### 2.8.2 Hematoxylin and eosin (H&E) staining

Pathological staining of paraffin-embedded tissue sections was performed using standard histological protocols. In brief, tissue sections were deparaffinized and rehydrated through a graded series of xylene and ethanol. Hematoxylin and eosin (H&E) staining was performed to assess overall intestinal architecture. For Gram staining, sections were sequentially incubated with crystal violet, Gram’s iodine, and safranin, followed by decolorization with a solvent-based decolorizer and subsequent treatment with picric acid–acetone. Following final dehydration through ethanol and xylene, sections were coverslipped using Fluoromount aqueous mounting medium (Sigma-Aldrich, Cat. #F4680). Images were captured using an upright wide-field Nikon Eclipse 90i microscope.

#### 2.8.3 Periodic acid–Schiff (PAS) staining

Paraffin-embedded tissue sections were deparaffinized and rehydrated through a graded series of xylene and ethanol solutions. For histological evaluation of intestinal architecture, sections were stained with hematoxylin and eosin (H&E). To visualize goblet cells and mucus production, periodic acid–Schiff–alcian blue (PAS-AB) staining was performed according to standard protocols. For bacterial staining, sections were subjected to Gram staining using crystal violet, Gram’s iodine, and safranin, with intermediate decolorization using an alcohol-based decolorizer, followed by a picric acid–acetone treatment. After staining, sections were dehydrated through graded alcohol and xylene, then mounted using Fluoromount aqueous mounting medium (Sigma-Aldrich, Cat. #F4680). Images were acquired using an upright wide-field Nikon Eclipse 90i microscope.

### 2.9 Statistical analysis

**FIGURE 1:**
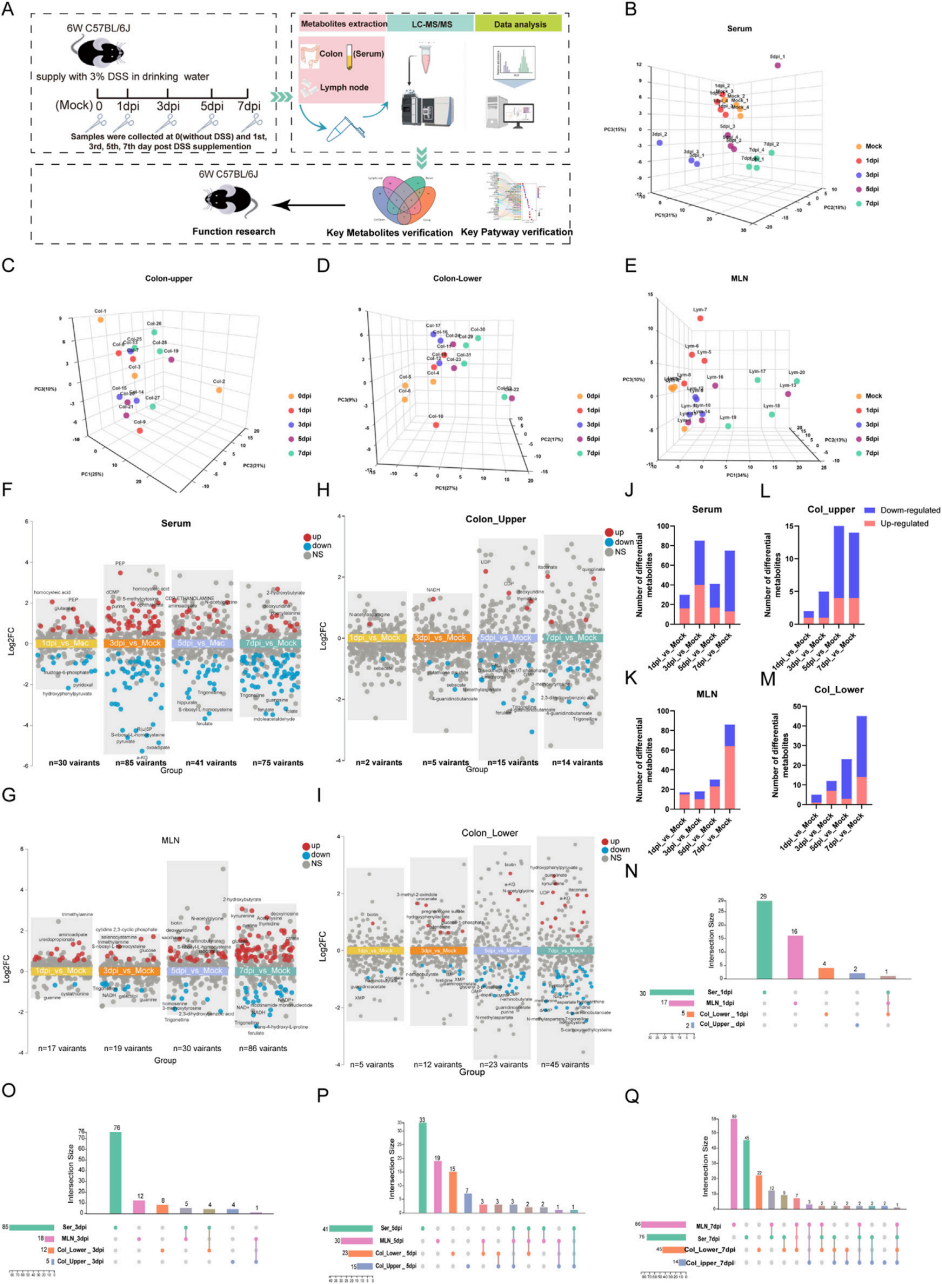
Temporal metabolomics reveals the severity of colitis during DSS exposure. **(A)** Schematic diagram of the study design. **(B–E)** Principal component analysis of metabolomic profiles from serum, upper colon, lower colon, and mesenteric lymph nodes (MLNs). **(F–I)** Multi-group volcano plots of serum, upper colon, lower colon, and MLN (|Log_2_ FC|>0.5, P-value <0.05). **(J–M)** Statistical results of upregulated and downregulated metabolites in serum, upper colon, lower colon, and MLN at 1 day, 3 days, 5 days, and 7 days post-DSS exposure (dpi). **(N–Q)** Interactive Venn diagrams showing the distribution of differential metabolites across serum, col_upper, col_lower, and MLNs on the 1st, 3rd, 5th, and 7th days post-DSS exposure (dpi).

Data processing procedures and statistics analysis: The MS data were collected using Analyst software (v.1.7.1, SCIEX), and the relative amounts of metabolites were analyzed using MultiQuant software (v.3.0.3, SCIEX). For metabolomics, quality control (QC) samples were prepared and inserted at an interval of every six samples to monitor the stability of the instrument and normalize the variation among samples and batch effects. statTarget was applied to data normalization and QC calibration. Statistical analysis was performed by normalization to the median intensity of all identified compounds and Pareto scaling. Then, the data were further corrected by internal standard and tissue weight. Principal component analysis (PCA) was used for multivariate statistics and visualization, specifically for outlier detection. The prcom and FactoMineR functions were used for calculating and plotting (Figures 1B–E).

For longitudinal trajectory clustering, fold change (FC) analyses were calculated with MetaboAnalystR, by using the mean of metabolite abundance ratios in each tissue, relative to the Mock group as the baseline. Then, one-way analysis of variance (ANOVA) was performed on data from different time points of DSS exposure. To quantify the results, we calculated the number of significantly changed metabolites (|log_2_ FC| > 0.5, P-value < 0.05) after DSS exposure, and soft clustering of the altered metabolites selected by ANOVA was performed using MFuzz (v2.54.0). Pathway enrichment analysis was performed using MetaboAnalystR with default parameters and Kyoto Encyclopedia of Genes and Genomes (KEGG) IDs. Quantitative enrichment analysis was performed via the Enrichment Analysis module using the pathway-based KEGG metabolite set library (84 metabolite sets based on human metabolic pathways). The VIP score was calculated by the MetaboAnalyst PLS-DA module.

The experimental results were presented as mean ± standard deviation (mean ± SD). An ANOVA test followed by Tukey’s HSD post-hoc test was used for the determination of the meaningful metabolites (Shimshoni et al., 2024). Data represent the mean ± standard error of the mean calculated using GraphPad Prism 8 software. For pairwise comparisons, t-tests were used. For comparisons between more than two groups, one-way analysis of variance (ANOVA) was performed. An adjusted P-value <0.05 was considered statistically significant.

## 3 Results

### 3.1 Temporal metabolomics reveals the severity of colitis during DSS exposure

To determine the temporal and systematic metabolic alteration during the development of colitis, we collected four tissues of DSS-induced mice colitis, including serum, mesenteric lymph nodes (MLNs), and upper and lower segments of the colon (col_upper and col_lower) at the 0 (Mock), 1st, 3rd, 5th, and 7th day post-DSS induction (dpi). These samples were then subjected to targeted metabolomic analysis by using an ultra-high-performance liquid chromatography coupled with triple quadrupole mass spectrometry (UHPLC-MS/MS). The study design is illustrated in Figure 1A. A total of 271 metabolites were reliably detected by the targeted metabolomics (Supplementary Table S1). PCA analysis revealed an increasing metabolic distance from the Mock group during the progress of colitis, especially in serum and the lower segment of the colon (col_lower) (Figures 1B–E). To explore the detailed metabolic dynamics during colitis progression, we compared the metabolites at each time point (1 dpi, 3 dpi, 5 dpi, and 7 dpi) with the Mock group. The multi-volcano plots highlight the differential metabolites at each time point after DSS exposure. Serum and MLNs exhibited the earliest and most pronounced metabolic disturbances, with 30 differential metabolites at 1 dpi and 86 variant metabolites at 7 dpi, respectively. The upper and lower segments of the colon displayed a growing metabolic disturbance as time went by, with significant metabolic disorders becoming evident by the fifth day (Figures 1F–I). Statistical analysis of differential metabolites revealed that the downregulated metabolites in serum, Col_upper, and Col_lower increased over time, suggesting colitis as a cachectic disease. On the other hand, the increasing number of upregulated metabolites in MLNs and col-lower indicate the progression of colitis (Figures 1J–M). We also examined the similarities and differences in metabolic changes across the four tissues. Serum exhibited the most distinctive metabolic alterations throughout the study. MLNs and both colon segments (upper and lower) shared a series of differential metabolites at 3 dpi, 5 dpi, and 7 dpi (Figures 1N–Q), highlighting the metabolic communication among these tissues. In short, we have constructed a temporal metabolic profile for DSS-induced colitis.

### 3.2 Systemic activation of metabolic pathways in response to DSS exposure

**FIGURE 2:**
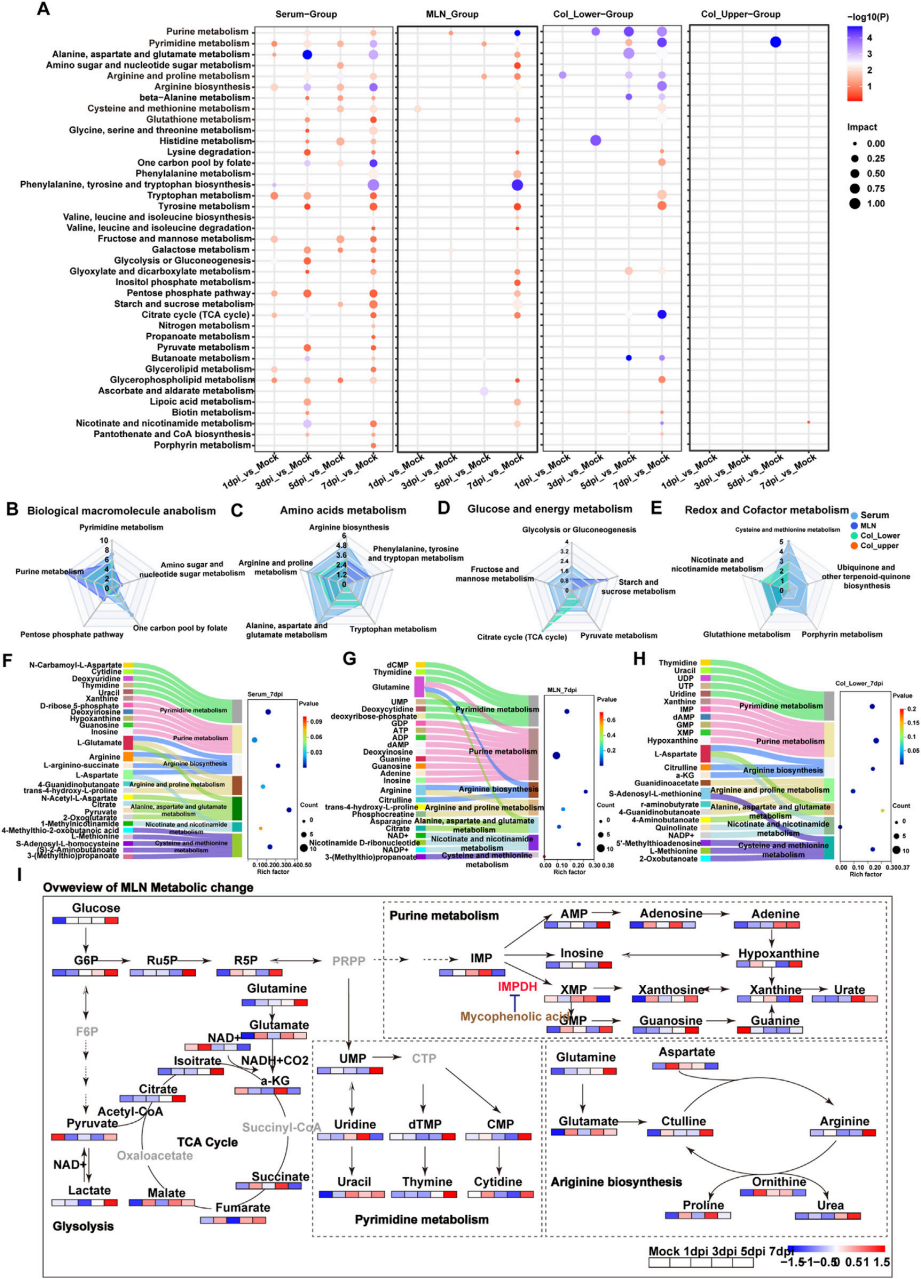
Systemic activation of metabolic pathways in response to DSS exposure. **(A)** KEGG enrichment analysis of differential metabolites in serum, MLNs, upper colon, and lower colon at 1 day, 3 days, 5 days, and 7 days post-DSS exposure (dpi). **(B–E)** Radar charts showing the number of altered metabolites in pathways related to **(B)** macromolecular biosynthesis, **(C)** amino acid metabolism, **(D)** glucose and energy metabolism, and **(E)** redox balance following DSS exposure. **(F–H)** Sankey bubble diagrams illustrating the relationship between differential metabolic pathways and their associated metabolites in serum, MLNs, and lower colon at day 7 post-DSS exposure. **(I)** Schematic depicting the key disturbed metabolic pathways in response to DSS exposure.

To identify the metabolic pathways directly impacted by DSS exposure or those exhibiting time-specific alterations, we performed Kyoto Encyclopedia of Genes and Genomes (KEGG) enrichment analysis on significantly altered metabolites identified by multi-volcano plots (Figure 2A, |log_2_ FC| > 0.5, P-value <0.05). Pathway enrichment also reflects the growing impact of DSS exposure on metabolic pathways in serum, MLNs, and the lower segments of the colon as aggravating colitis. Notably, pyrimidine metabolism, tryptophan metabolism, glycerophospholipid metabolism, arginine biosynthesis, the citrate cycle, and the pentose phosphate pathway were particularly sensitive to DSS stimulation, with alterations observed in serum as early as day 1 post-DSS exposure. Purine metabolism exhibited an early disturbance in both the MLNs and the lower segment of the colon. Ultimately, DSS exposure elicited a robust enrichment of pathways involved in nucleotide metabolism (purine and pyrimidine) and AA metabolism (including arginine, proline, alanine, aspartate, and glutamate, arginine biosynthesis, cysteine, methionine, glycine, serine, threonine, phenylalanine, tyrosine, and tryptophan) in serum, MLNs, and Col-lower at 7 dpi (Figure 2A).

**FIGURE 3:**
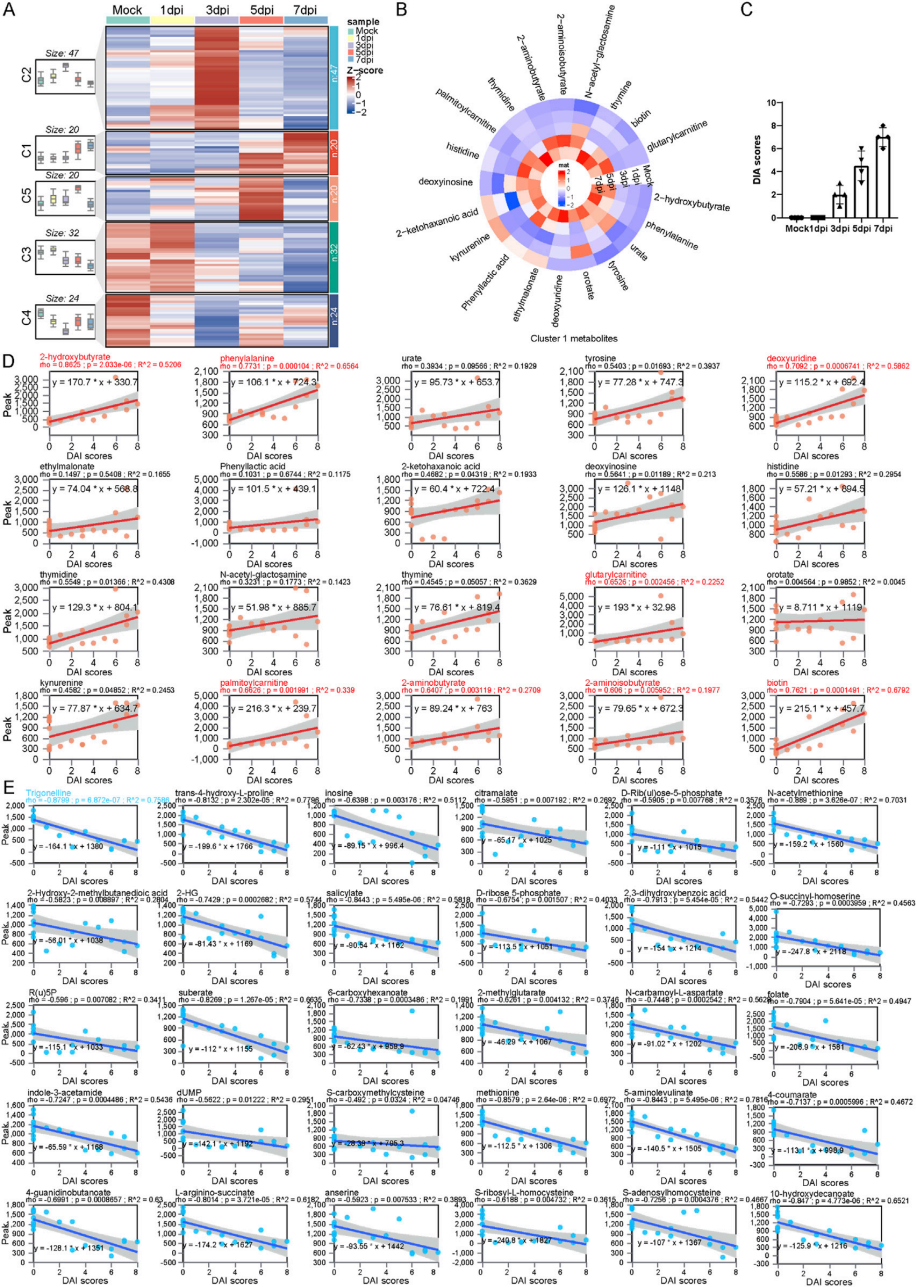
Trajectory analysis determines a series of serum metabolic biomarkers related to the severity of colitis. **(A)** Longitudinal trajectory clustering of significantly changed serum metabolites: black lines represent the average trajectory for each cluster; the heatmap shows the relative abundance of metabolites in each sample. **(B)** Heatmap of the relative abundance of cluster 1 metabolites. **(C)** Statistical bar chart of DAI scores at different time points following DSS exposure. **(D)** Spearman correlation analysis between the relative abundance of the cluster 1 metabolites and DAI scores. **(E)** Spearman correlation analysis between the relative abundance of the cluster 3 metabolites and DAI scores.

In order to quantify the number of altered metabolites in these metabolic pathways, pathways were divided into four functional groups, including biological macromolecule anabolism, amino acids, glucose and energy, and redox and cofactor metabolism. Serum exhibited the greatest number of disturbances in one carbon pool by folate, amino acids, and purine metabolism, which can provide biomolecules for cell proliferation, suggesting the systemic activation of the immune response. This was further supported by the altered purine metabolism and tricarboxylic acid (TCA) cycle in MLNs. The redox balance is crucial for tissue adaptation to stress, and radar plot analysis revealed the most significantly altered redox-related pathways, including cysteine and methionine metabolism in serum, glutathione metabolism in both upper and lower segments of the colon, and nicotinate and nicotinamide metabolism in the MLNs, upper colon, and lower colon (Figures 3B–E). Overall, these data demonstrated the local and immune-related metabolic changes that occur during DSS exposure. To better illustrate the relationship between metabolites and metabolic pathways, we used Sankey diagrams based on KEGG pathway enrichment analysis. We found that purine metabolism in the MLNs was the most significantly enriched pathway at 7 dpi; the increased purine metabolism may be a symbol of immune cell proliferation in the MLNs (Figures 2F–H). To further investigate the metabolic changes of immune cells in the MLNs, we mapped metabolites related to energy metabolism, macromolecular biosynthesis, and amino acid metabolism into metabolic pathways (Figure 2I). After DSS stimulation, the MLNs exhibited an increased demand for glucose; however, the enhanced glucose uptake did not increase glycolysis but instead fluxed into purine metabolism via the pentose phosphate pathway (PPP), particularly boosting the production of the intermediate metabolite xanthine. Recent studies have shown that serum xanthine levels are elevated in patients with diarrhea-predominant irritable bowel syndrome (IBS-D), and xanthine injection can mimic IBS-D symptoms in mice [PMID: 39366386]. Our data further demonstrated that xanthine derived from the MLNs may contribute to the pathogenesis of IBD. Additionally, the rapid proliferation of immune cells in the MLNs increases the demand for energy, primarily supplied by the tricarboxylic acid (TCA) cycle through glutamate, leading to an imbalance in redox homeostasis in the MLNs. Therefore, purine metabolism may be a potential therapeutic target for colitis treatment.

### 3.3 Trajectory analysis determines a series of serum metabolic biomarkers related to the severity of colitis

To further identify the dynamic features of serum metabolite changes at longitudinal stages, we conduct a c-means clustering analysis on differential metabolite data from serum during DSS exposure. A total of 143 differential metabolites were categorized into five main clusters, each exhibiting distinct patterns. Metabolites enriched in cluster 1 increased gradually, followed by a sharp rise at 5 dpi. Cluster 2 contained metabolites that increased quickly and peaked at 3 dpi, then declined gradually; cluster 3 metabolites exhibited a sustained decrease after DSS exposure; molecules in cluster 4 displayed a “v” shape trend, while metabolites in cluster 5 increased initially and became exhausted in the later stage (Figure 3A). Notably, metabolites in cluster 1 followed a trend similar to that of the disease activity index (DAI) score for colitis; in contrast, cluster 3 metabolites exhibit an opposite trend compared to metabolites in cluster 1 (Figures 3B,C). We performed a Spearman correlation analysis between cluster 1, cluster 3 metabolites, and DAI scores. Linear regression analysis showed that the level of cluster 1 metabolites was positively correlated with DAI scores. The correlation coefficient among metabolites such as 2-hydroxybutyrate, phenylalanine, deoxyuridine, glutarylcarnitine, palmitoylcarnitine, 2-aminobutyrate, and DAI scores was greater than 0.6, indicating that these metabolites could serve as potential serum biomarkers for colitis progression (Figure 3D). On the other hand, a comprehensive negative correlation was observed between DAI scores and cluster 3 metabolites. Trigonelline showed the greatest coefficient among these metabolites (Figure 3E).

### 3.4 Trigonelline was the most important metabolite during colitis progression

**FIGURE 4:**
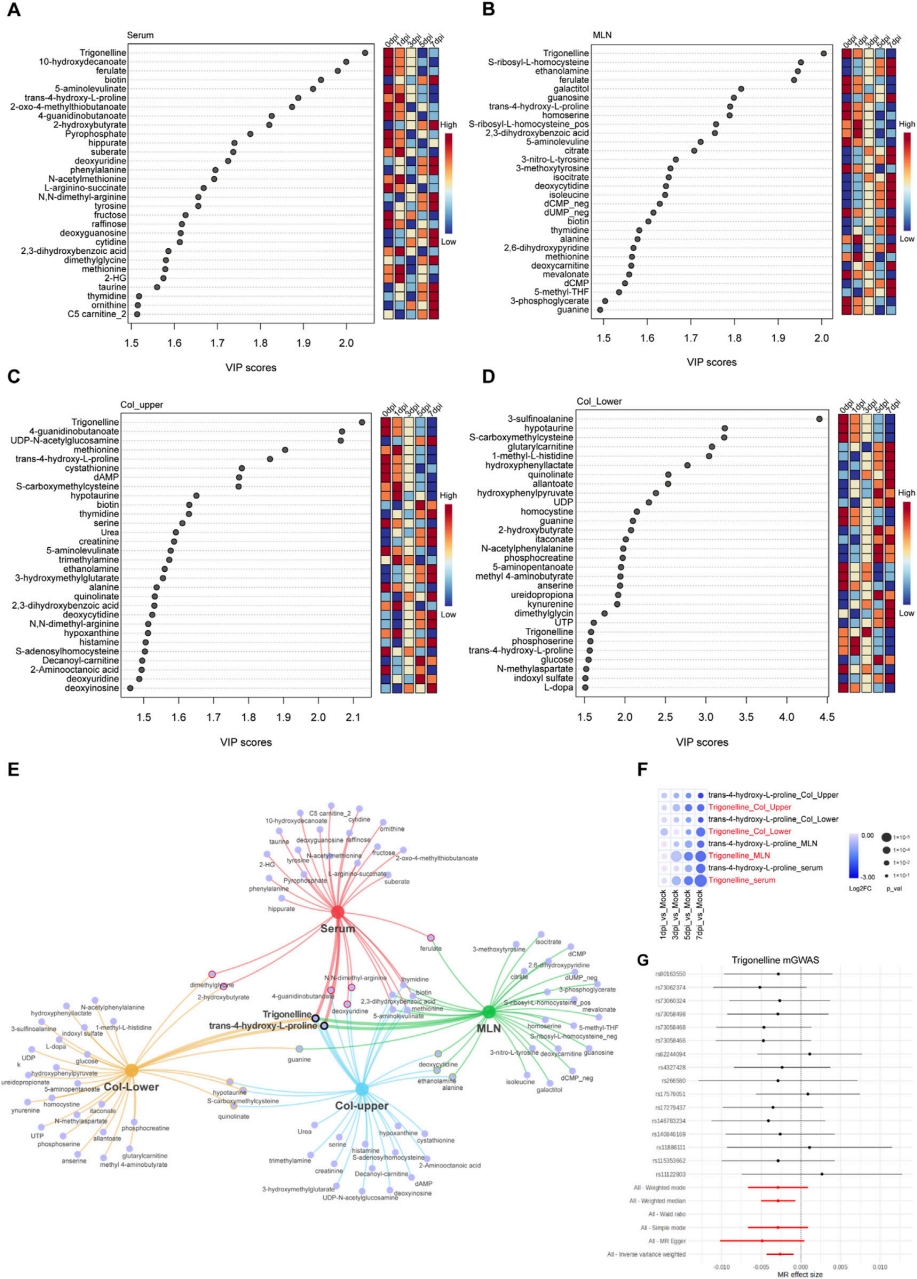
Trigonelline was the most important metabolite during colitis progression. **(A–D)** Top 30 metabolites based on VIP scores in serum **(A)** MLNs **(B)** upper colon **(C)** and lower colon **(D)** following DSS exposure. **(E)** Network Venn diagram showing the most significantly affected metabolites in various organs after DSS exposure. **(F)** Bubble heatmap illustrating the temporal changes of trigonelline and trans-4-L-proline at different time points post-DSS exposure. **(G)** mGWAS analysis of trigonelline with the colorectal polyp database.

To determine the most important metabolites during the progression of DSS-induced colitis, we performed VIP analysis on metabolites in four tissues. We found that trigonelline and trans-4-hydroxy-L-proline were significantly altered across all four tissues and organs, suggesting that these two metabolites may be closely associated with the pathogenesis of colitis (Figures 4A–D). Additionally, in the upper and lower segments of the colon, hypotaurine, S-carboxymethylcysteine, and quinolinate exhibited significant changes. Hypotaurine, a precursor of taurine, decreased gradually after DSS exposure; s-carboxymethylcysteine, a metabolite involved in mucus activity regulation in chronic obstructive pulmonary disease (COPD), and quinolinate, a tryptophan metabolite and NAD precursor, also displayed significant changes (Figures 4C–E). These findings suggest that reduced antioxidant capacity in the colon occurs after DSS exposure. Furthermore, the significant downregulation of trigonelline and trans-4-hydroxy-L-proline in multiple organs after DSS exposure suggests the potential therapeutic function of these molecules (Figure 4F). We further conducted mGWAS analysis of trigonelline and trans-4-hydroxy-L-proline with published datasets on genes related to colon polyps and found that trigonelline is closely linked to multiple single-nucleotide polymorphisms (SNPs) of colon polyps, suggesting that it may be the most promising target for colitis treatment (Figure 4G).

### 3.5 Therapeutic effects of trigonelline and mycophenolic acid on DSS-induced colitis in mice

**FIGURE 5:**
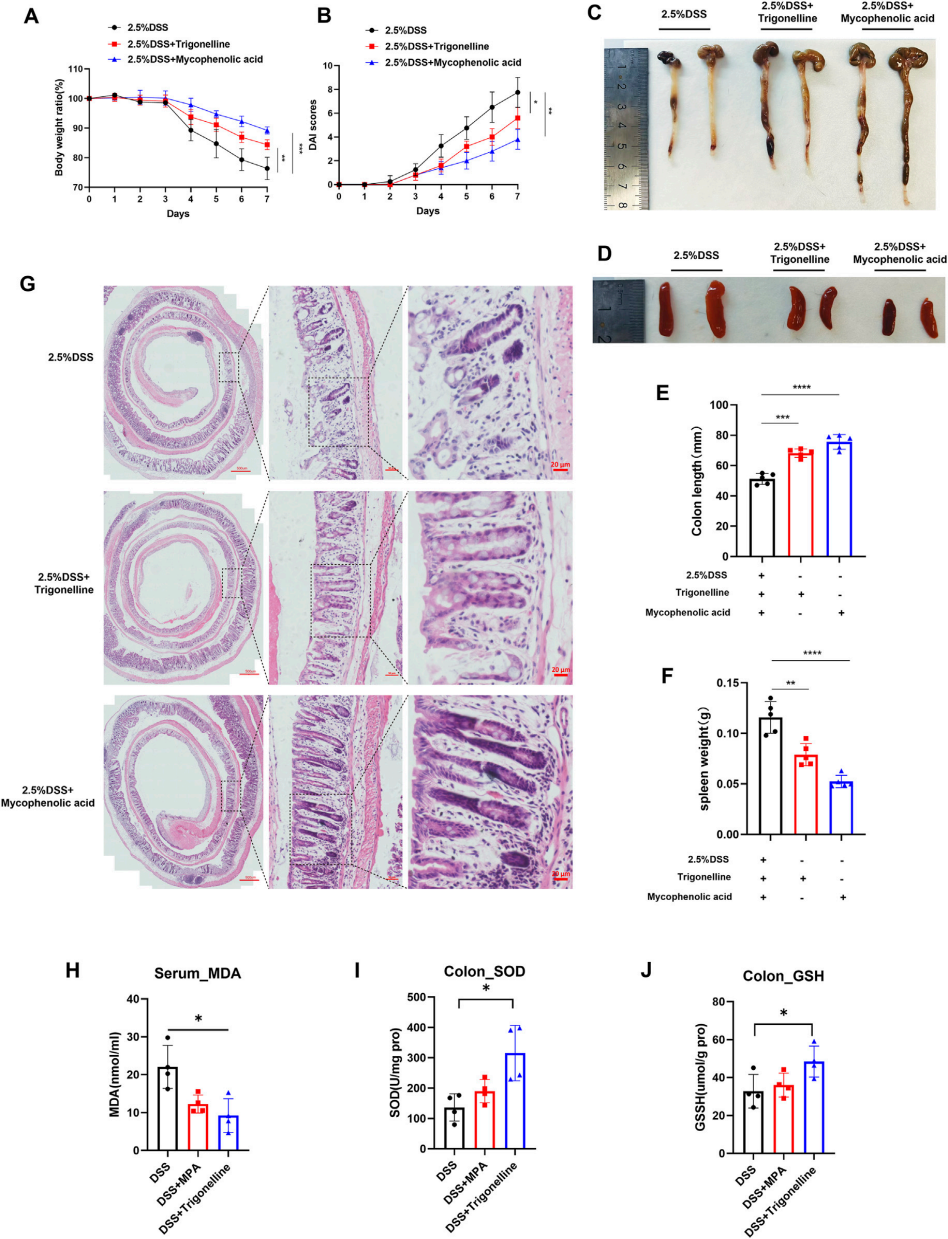
Therapeutic effects of trigonelline and MPA on DSS-induced colitis in mice. **(A)** Body weight changes in mice throughout DSS treatment with or without trigonelline and MPA intervention. **(B)** Disease activity index (DAI) scores reflect the clinical severity of colitis. **(C–D)** Representative images and quantification of colon lengths across experimental groups. **(E–F)** Representative images and quantification of spleen sizes in treated and untreated groups. **(G)** Representative H&E-stained colonic tissue sections illustrating mucosal integrity and inflammatory cell infiltration. **(H–J)** Serum MDA levels and colonic tissue SOD and GSH concentrations, demonstrating oxidative stress modulation by trigonelline and MPA treatment. Data represent mean ± SD; *P < 0.05, **P < 0.01, and ***P < 0.001 compared to the DSS-only group.

Based on our earlier metabolomic data, we identified trigonelline as a crucial metabolite linked to colitis progression and noted significant enrichment in purine metabolism pathways, suggesting an intense immune response during inflammation. To further investigate the therapeutic effects of trigonelline and the purine metabolism inhibitor MPA, we established a mouse colitis model by administering 2.5% DSS in drinking water. After DSS induction, mice exhibited rapid colonic shortening, intestinal stiffness, transition from loose to watery stools, absence of solid fecal pellets, and visible rectal bleeding. Combined administration of trigonelline and MPA significantly improved fecal consistency and alleviated rectal bleeding, exhibiting superior therapeutic efficacy compared to DSS-only controls. Body weight loss was significantly attenuated in the treated groups (Figure 5A). Disease activity index (DAI) scores confirmed that the treated mice displayed notably improved clinical conditions (Figure 5B). The intervention groups showed statistically significant restoration of colon length and reduced spleen size compared to DSS-treated mice (Figures 5C–F). Histological analysis via hematoxylin and eosin (H&E) staining revealed severe mucosal damage, disrupted glandular architecture, loss of crypt structures, extensive ulceration, caseous necrosis, disorganized gland arrangement, significant goblet cell depletion, and pronounced lymphocytic and neutrophilic infiltration in DSS-treated mice. However, treatment with trigonelline and MPA effectively reduced mucosal injury, preserved glandular structure, maintained goblet cell populations, and minimized inflammatory cell infiltration (Figure 5G). Biochemical assays demonstrated that trigonelline and MPA treatments significantly decreased serum malondialdehyde (MDA) levels and elevated colonic superoxide dismutase (SOD) and glutathione (GSH) contents, indicating reduced oxidative stress and enhanced antioxidative capacity (Figures 5H–J).

### 3.6 Trigonelline and MPA restore intestinal immune homeostasis and increase NAD^+^ levels in DSS-induced colitis

**FIGURE 6:**
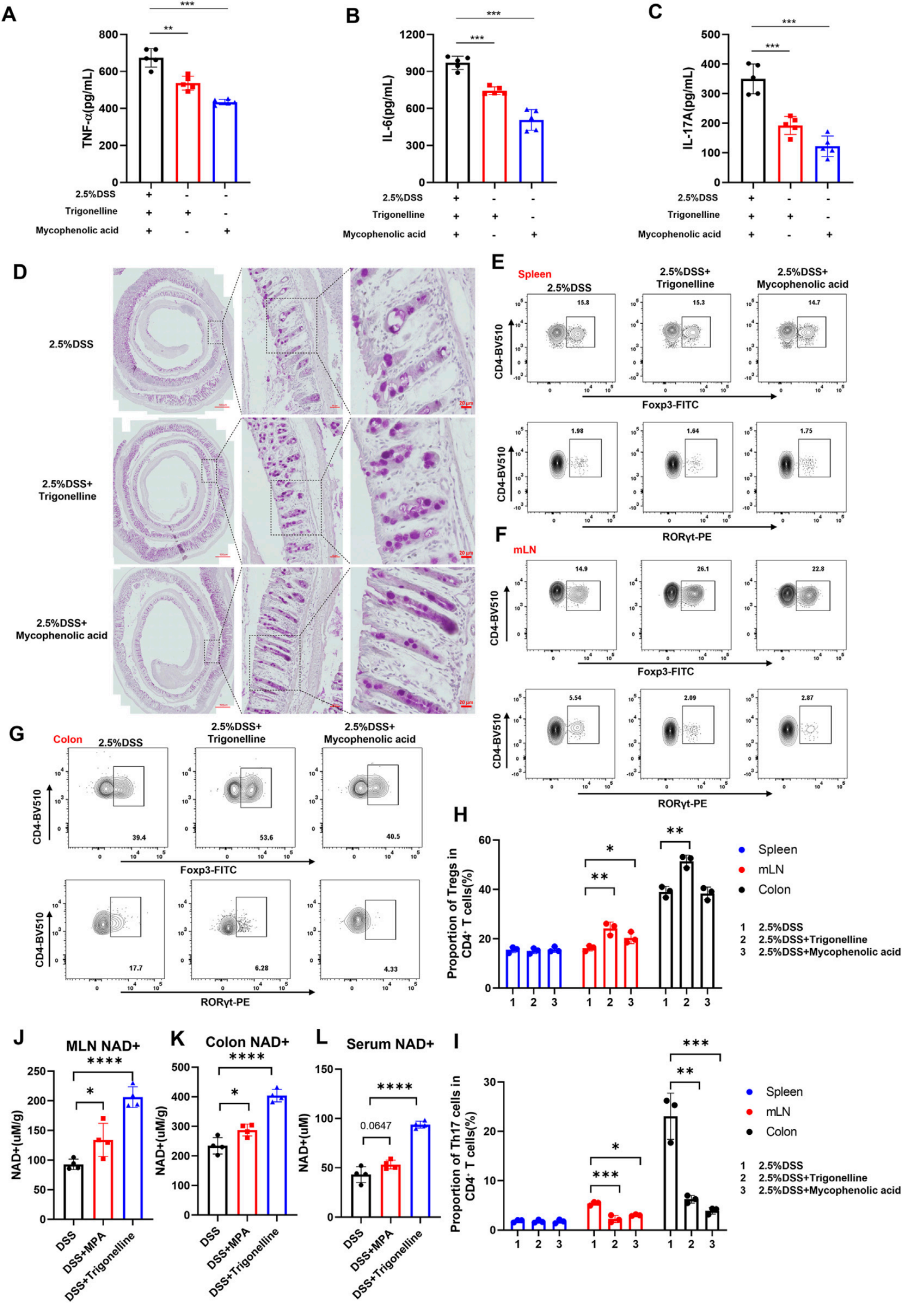
Trigonelline and MPA restore intestinal immune homeostasis and increase NAD^+^ levels in DSS-induced colitis. **(A–C)** ELISA measurements showing significantly reduced expression of pro-inflammatory cytokines TNF-α **(A)**, IL-6 **(B)**, and IL-17A **(C)** in colon tissues after trigonelline and MPA treatment. **(D)** Representative PAS-stained colon sections demonstrating restored goblet cell number and morphology following treatment. **(E–I)** Flow cytometric analysis illustrating increased CD4^+^Foxp3^+^ Treg frequencies and decreased CD4^+^RORγt^+^ Th17 frequencies in mesenteric lymph nodes and colonic lamina propria, with no significant change in the spleen. **(J–L)** NAD^+^ content quantification in mesenteric lymph nodes, colonic tissues, and serum, showing significantly increased levels after treatment. Data are shown as mean ± SD; *P < 0.05, **P < 0.01, and ***P < 0.001 compared to DSS-only controls.

To investigate the anti-inflammatory effects of trigonelline and the purine metabolism inhibitor MPA on DSS-induced colitis, we assessed pro-inflammatory cytokine levels in colonic tissues using ELISA. The results demonstrated that treatment with trigonelline and MPA significantly reduced the expression of TNF-α (Figure 6A), IL-6 (Figure 6B), and IL-17A (Figure 6C), compared to the DSS-only group. Periodic acid–Schiff (PAS) staining revealed a notable loss and morphological abnormality of goblet cells following DSS exposure, indicative of disrupted epithelial differentiation. Notably, co-treatment with trigonelline and MPA restored goblet cell numbers and preserved their typical morphology (Figure 6D).

Given the pivotal role of regulatory T (Treg) and Th17 cells in maintaining immune homeostasis and tolerance, we next examined the impact of trigonelline and MPA on their distribution. Flow cytometric analysis of lymphocytes isolated from mesenteric lymph nodes (MLNs), spleen, and colonic lamina propria showed that trigonelline and MPA significantly increased the frequency of CD4^+^Foxp3^+^ Tregs while reducing the proportion of CD4^+^RORγt^+^ Th17 cells in MLNs and the colonic lamina propria but not in the spleen (Figures 6E–I). These findings suggest a correction of the Th17/Treg imbalance, which is known to be critical in the pathogenesis of inflammatory bowel disease (IBD). By modulating these adaptive lymphocyte subsets, trigonelline and MPA appear to re-establish intestinal immune homeostasis and attenuate inflammatory responses. NAD^+^ levels were quantified in the MLNs, colon, and serum. Notably, treatment with trigonelline and MPA significantly increased NAD^+^ concentrations across all examined tissues (Figures 6J–L), suggesting enhanced cellular metabolic function and immunoregulatory potential.

### 3.7 Trigonelline and MPA reduce Treg and Th17 cell infiltration in both proximal and distal colon segments of DSS-treated mice

**FIGURE 7:**
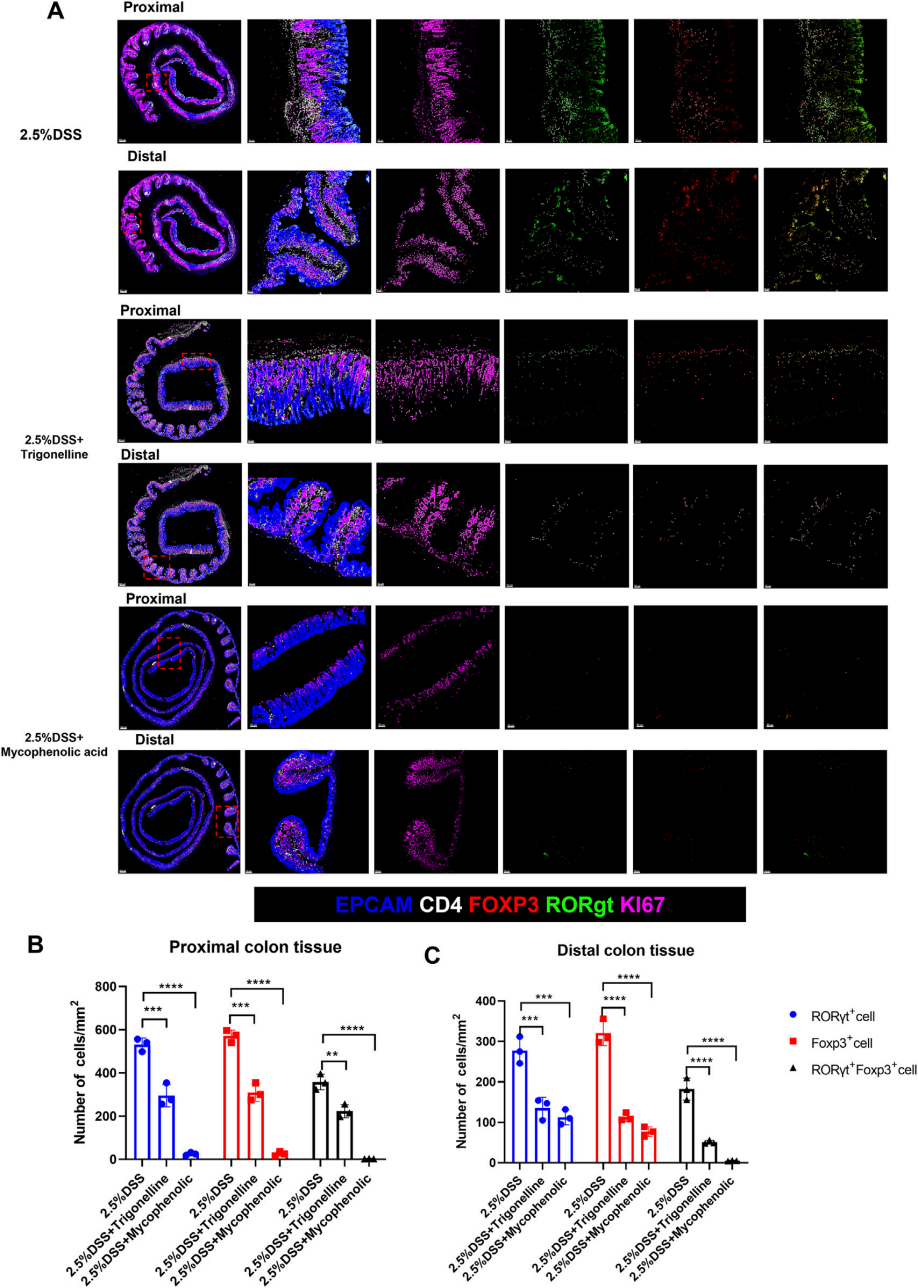
Trigonelline and MPA reduce Treg and Th17 cell infiltration in both proximal and distal colon of DSS-treated mice. **(A)** Representative immunofluorescence images of proximal and distal colon sections stained for CD4^+^Foxp3^+^ Tregs, CD4^+^RORγt^+^ Th17 cells, and Foxp3^+^RORγt^+^ double-positive cells in each treatment group (scale bar, 50 µm). **(B,C)** Quantification of infiltrating Treg and Th17 cells per high-power field in proximal **(B)** and distal **(C)** colon. Data are shown as mean ± SD. *P < 0.05, **P < 0.01, and ***P < 0.001 versus the DSS group.

To further visualize the impact of trigonelline and mycophenolic acid (MPA) on immune cell infiltration in distinct colonic regions of DSS-induced colitis, we performed immunofluorescence staining on both proximal and distal colon sections. Notably, combined treatment with trigonelline and MPA markedly reduced the infiltration of Treg cells, Th17 cells, and Foxp3^+^RORγt^+^ double-positive cells across both regions compared to DSS-only controls (Figure 7A). Quantitative analysis confirmed significant reductions in the numbers of infiltrating Treg and Th17 cells (Figures 7B,C), suggesting that trigonelline and MPA effectively suppress mucosal immune activation in a region-independent manner.

## 4 Discussion

In the current study, we employed a comprehensive temporal metabolomic approach in a DSS-induced colitis mouse model, systematically elucidating metabolic shifts in colonic tissues, mesenteric lymph nodes, and serum at multiple critical time points during disease progression. Our findings provide new insights into the temporal metabolic remodeling that occurs during intestinal inflammation and highlight the critical role of purine and tryptophan metabolisms in shaping the mucosal immune response. Systematic metabolomic profiling revealed that both the purine and tryptophan pathways are dynamically regulated across different anatomical compartments during DSS-induced colitis, with distinct changes observed between the acute and chronic phases. Specifically, we observed a significant decline in serotonin levels and a concomitant accumulation of kynurenine in the colon and MLNs, suggesting an inflammation-driven metabolic reprogramming toward the kynurenine pathway that may promote immune dysregulation.

Through metabolomic profiling, we identified a series of serum metabolites significantly associated with colitis severity. Notably, trigonelline emerged as a pivotal biomarker correlating with disease progression. Previous studies indicated that trigonelline possesses anti-inflammatory properties, supporting its relevance in inflammatory conditions (Shajib and Khan, 2015). Consistent with this, our experimental validation further demonstrated that trigonelline supplementation significantly mitigated inflammation, reduced pathological damage, and improved immune cell infiltration within colonic tissues and mesenteric lymph nodes. Furthermore, metabolic pathway enrichment across multiple tissues revealed purine metabolism as a prominently activated pathway during the inflammatory response, particularly in colonic regions and lymph nodes. This robust activation likely reflects increased nucleotide demand for rapidly proliferating immune cells during inflammation (Wang et al., 2024; Pålsson-McDermott and O’Neill, 2020).

Purines are critical mediators of inflammation, immune cell proliferation, and differentiation, thus making purine metabolism a promising therapeutic target in inflammatory conditions (Linden et al., 2019; Gu et al., 2021; Furuhashi, 2020). Indeed, previous studies have demonstrated the efficacy of purine metabolism inhibitors such as mycophenolic acid (MPA) in attenuating inflammatory responses in various disease models (Lovelace et al., 2017; Kasahara et al., 2023; Hotamisligil, 2006). In our study, trigonelline and MPA were found to modulate metabolic pathways that are closely linked to the amelioration of colitis. The altered metabolites, particularly purine metabolism intermediates and trigonelline, play a crucial role in regulating inflammation. Purine metabolism is essential for immune cell activation, and we observed that its activation in colonic tissues and mesenteric lymph nodes (MLNs) correlates with enhanced immune responses. Specifically, metabolites like adenosine influence key inflammatory pathways, such as NF-κB and JAK/STAT, which are involved in immune cell function and inflammation.

Our results corroborate these findings, showing that MPA intervention notably alleviated inflammatory symptoms in DSS-treated mice. MPA effectively reduced pathological inflammation and improved the immune microenvironment by attenuating immune cell infiltration and restoring the Th17/Treg balance, which is frequently disrupted in IBD (Heintzman et al., 2022; Chen et al., 2022). Th17 cells have been closely associated with exacerbation of intestinal inflammation, whereas Treg cells are pivotal in maintaining intestinal immune homeostasis and preventing excessive inflammation (Adams et al., 2024; Zaiatz Bittencourt et al., 2021; Shi et al., 2025). The rebalancing of Th17/Treg populations following MPA treatment further validates the therapeutic potential of targeting purine metabolism pathways to modulate intestinal immune responses. Interestingly, the combined use of trigonelline and MPA provided synergistic therapeutic effects, indicating a complex interplay between metabolic pathways in modulating immune responses. Trigonelline likely acts by stabilizing the intestinal epithelial barrier and inhibiting pro-inflammatory cytokine release, thus complementing the immune-regulatory effects of MPA (Anwar et al., 2018). These observations highlight the importance of integrating multiple metabolic modulators to achieve comprehensive therapeutic outcomes.

Our study also provides novel insights into the temporal dynamics of metabolic activation throughout the progression of colitis. The systematic metabolic changes detected from day 1 to day 7 following DSS administration reflect an evolving metabolic environment responsive to inflammatory cues. Recent literature emphasizes that inflammatory states significantly reshape metabolic pathways, including glycolysis, amino acid metabolism, and nucleotide synthesis, facilitating immune cell proliferation and cytokine production (Shimshoni et al., 2024; Hu et al., 2024; Jung et al., 2019; Chauhan and Saha, 2018).

Despite these significant findings, our study has some limitations. The precise mechanisms underlying the effects of trigonelline and MPA on immune cell populations require further investigation. Future studies should focus on clarifying the molecular mechanisms by which trigonelline modulates purine metabolism and immune responses. Moreover, translational studies evaluating the efficacy and safety of these metabolic interventions in clinical IBD settings are warranted.

Based on our data, we propose that metabolic regulation attenuates intestinal inflammation through the activation of purine metabolism and immune modulation. Both trigonelline and MPA individually enhance purine metabolism, which influences key inflammatory pathways such as NF-κB and JAK/STAT. These pathways regulate immune cell activation and cytokine production. Additionally, both treatments restore the Th17/Treg balance, promoting immune tolerance. Together, these mechanisms suppress pro-inflammatory signaling, reduce immune cell infiltration, and alleviate histopathological damage, ultimately attenuating colitis.

In conclusion, our study advances the understanding of metabolic reprogramming in IBD pathogenesis, emphasizing trigonelline and purine metabolism as critical modulators of intestinal inflammation. The therapeutic potential demonstrated by interventions targeting these pathways opens new avenues for innovative treatment strategies aimed at metabolic regulation in IBD.

## Data Availability

The original contributions presented in the study are included in the article/Supplementary Material; further inquiries can be directed to the corresponding authors.
